# Rapid adulteration detection of cold pressed oils with their refined versions by UV–Vis spectroscopy

**DOI:** 10.1038/s41598-020-72558-7

**Published:** 2020-09-30

**Authors:** Simona Popa, Marius Silviu Milea, Sorina Boran, Sabina Violeta Nițu, Giannin Emanuel Moșoarcă, Cosmin Vancea, Radu Ioan Lazău

**Affiliations:** Faculty of Industrial Chemistry and Environmental Engineering, Politehnica University of Timisoara, Bd. V. Parvan No. 6, 300223 Timisoara, Romania

**Keywords:** Nutrition, Chemistry

## Abstract

The aim of this study is the rapid detection of food pressed oils adulteration with their refined versions, using UV–Vis spectroscopy. The study investigates some common oil physico-chemical parameters such are: density, viscosity, refractive index, acid index, peroxide value, saponification index, to detect differences between cold pressed oils versus refined ones, for some food-grade oils found on Romanian market, as well as FT-IR spectroscopy and GC–MS analytical method, obtaining similar results to those presented in the literature data. The difference between some of the obtained results is not relevant for telling the cold-pressed oils from their refined version for adulteration investigation purpose. Colour analysis instead is a very good method to differentiate a cold pressed oil from a refined one. Taking this into account, the cold pressed oils and their refined versions were mixed in different proportions, and their colour properties were analyzed, obtaining linear dependences for a* and b* CIE L*a*b* parameters with cold pressed oil content in the mixture. Dependence equations were proposed.

## Introduction

Beans and seeds are the most important vegetable oil sources. Some oils are obtained from cold pressing technology, which is environmentally friendly, preserves the nutrients in the oils and is and easy to perform. Cold pressed oils contain natural ingredients with numerous health benefits. There are different methods to obtain refined oils in food industry. By refining technologies, the undesirable materials may be removed along with some valuable components^1,2^. Quality differences of cold pressed vs. refined oils was reported^3,4^. Some physico-chemical parameters, such are: density, acid index, peroxide value, viscosity, or GC–MS (gas chromatography mass spectrometry) detection may be used to distinguish a cold pressed oil from a refined one^3^. Fatty acids composition of vegetable oils^5–9^, influences the human health, lipids being among fundamental nutrients^10^.

Price of food oils depends on their quality and purity, cold pressed oils being more expensive, that is why labels on oil bottles must mention whether it is a crude or a refined oil. Cold pressed oils are frequently subjected to fraud by mixing with different other seeds oils with inferior quality. Standards and rules are introduced by international quality control organizations to detect and prevent food oil falsification^11^. Some authors proposed methods to investigate these adulterations, such are: three-dimensional fluorescence spectroscopy^12^, UV–IMS and chemometric analysis^13^, fluorescence quenching method with aqueous CTAB-coated quantum dots^14^, confocal X-ray scattering analysis with coherent/incoherent scattered X-rays^15^, near-infrared spectroscopy and chemometric techniques^16^, optical thin-film biosensor chips^17^, or stimulated Brillouin scattering in combination with visible absorption spectroscopy^18^.

Oil colour is an important property for consumers^19^ and a good indicator of oil quality. Yet, this feature was presented only in few articles^7,20^.

To the best of our knowledge, there are no reports on detecting adulteration of cold pressed oils with their refined versions, or the use of CIEL*a*b* colour space in UV–Vis spectroscopy method for adulteration detection.

The purpose of this paper is to highlight that UV–Vis spectroscopy is a rapid and facile method for detecting adulteration of some cold pressed oils with their refined versions in different proportions, comparing to some other known and used methods.

## Results and discussion

### Physico-chemical parameters

Table 1 shows that physico-chemical parameters regarding density, viscosity, acid index, peroxide value, saponification index for the oils are similar to other results presented in literature^3,4,7,20–24^. Refractive indexes differ only for the last decimal between the cold pressed oils and the refined ones, being higher for the refined oils, all the other physico-chemical parameters are higher for cold pressed oils.

**Table 1:** Physico-chemical properties of the cold pressed (CP) and the refined (R) oils.

	Density, 20 °C g/cm^3^	Viscosity, cP, at 25 °C	Refractive index, at 25 °C	Acid index, mgKOH/g	Peroxide value, mEqO_2_/kg	Saponification index, mgKOH/g
**Coconut oil**						
CP	0.921^a^	0.446^a^	1.449^a^	1.23 ± 0.07	7.89 ± 0.08	266 ± 3
R	0.918^a^	0.367^a^	1.450^a^	0.50 ± 0.04	5,76 ± 0.05	256 ± 2
**Sunflower oil**						
CP	0.919	0.508	1.476	0.99 ± 0.02	4.95 ± 0.04	192 ± 3
R	0.917	0.497	1.477	0.60 ± 0.03	2.15 ± 0.03	188 ± 2
**Grapeseed oil**						
CP	0.929	0.464	1.478	1.86 ± 0.04	6.38 ± 0.06	192 ± 1
R	0.926	0.443	1.479	0.62 ± 0.02	2.82 ± 0.02	189 ± 2
**Canola oil**						
CP	0.921	0.563	1.474	1.32 ± 0.03	1.75 ± 0.04	191 ± 2
R	0.915	0.546	1.475	0.48 ± 0.02	0.68 ± 0.02	187 ± 1

^a^Values measured at 40 °C.

The results being very close to one another, and requiring time and chemical reagents, these physico-chemical parameters may not be used successfully for distinguishing a cold pressed oil from a refined one, and for the adulteration detection purposes.

### *FT-IR* spectra

*FT-IR* spectral correlations for different oils are not reported in the literature. That is why we wanted to emphasize if this analysis is suitable for differentiating the cold-pressed and refined oils. The results of FT-IR spectral analyses for the cold pressed and refined oils are as follows:

*Coconut oil* is a rich source of short- and medium-chain saturated fatty acids account for 70% of these fatty acids and it has a low content of unsaturated fatty acids with a negligible content of both ω-6 and ω-3 polyunsaturated fatty acids^7^.

Due to the low content of oleic acid (< 5%) and linoleic acid (< 1%), in the coconut oil, the carbon–carbon double bonds characteristic of the FT-IR spectroscopy at 1654 cm^−1^ assigned to stretching vibration ν_C=C_ could not be revealed. However, a small shoulder may be observed at 3,008 cm^−1^ that can be assigned to stretching vibration of the olefinic carbon–hydrogen bond ν_C-H_^25,26^.

*Sunflower oil* are rich in mono- (19.5%) and polyunsaturated (65.7%) fatty acids^3,7^. The FT-IR spectra of sunflower oils present the same characteristic bands of glycerol fatty saturated esters found in the coconut.

*Grapeseed oil* are rich in mono- (16.1%) and polyunsaturated (69.9%) fatty acids.

*Canola oil* are rich in mono- (63.3%) and polyunsaturated (28.1%) fatty acids^27^.

The FT-IR spectra of sunflower, grapeseed and canola oils present the same characteristic bands of glycerol fatty saturated esters found in the coconut.

- Characteristic bands for esters groups: intense band at 1746–7 cm^−1^ assigned to stretching vibration of the carbon–oxygen double bond ν_C=O_ in aliphatic esters; medium band at 1237–8 and 1163 cm^−1^ assigned to stretching vibration of the carbon–oxygen single bond ν_O=C–O_ in aliphatic esters; medium band at 1099–1119 cm^−1^ assigned to stretching vibration of the aliphatic tetrahedral carbon–oxygen bond ν_C-O_ in aliphatic esters^25,26^.
- Characteristic bands for alkyl groups (CH_2_ and CH in glycerol; CH_2_ and CH_3_ in saturated fatty acids): intense bands at 2924–7 cm^−1^ and a shoulder at 2953–5 cm^−1^ assigned to antisymmetric/symmetric stretching vibration of the aliphatic tetrahedral carbon–hydrogen bond ν^as^_C–H_ and 2854–5 cm^−1^ ν^s^_C–H_ aliphatic; medium bands at 1463–5 cm^−1^ and a weak band at 1416–9 cm^−1^ assigned to a antisymmetric bending in-plane deformation of the aliphatic tetrahedral carbon–hydrogen bond δ^as^_C-H_, and at 1376 cm^−1^ assigned to a symmetric bending in-plane deformation of the aliphatic tetrahedral carbon–hydrogen bond δ^s^_C–H_ aliphatic; the weak bands at 1031–4, 962–7, 912 cm^−1^ are not characteristic ones, being attributed to stretching vibration of the aliphatic tetrahedral carbon–carbon bond, ν_C-C_, to skeletal vibrations for aliphatic groups, and a medium band at 722–3 cm^−1^ assigned to scissoring out-of-plane deformation for the aliphatic groups CH_2_, CH_3_ and CH, γ_CH_^26,28^.
- In addition, the absorption bands characteristic of (*cis*) carbon–carbon double bonds of mono- and polyunsaturated fatty acids present in higher concentrations are also highlighted: weak-medium band at 3007–9 cm^−1^ assigned to stretching vibration of the olefinic carbon–hydrogen (=CH–) bond ν_C-H_; weak band at 1652–4 cm^−1^ assigned to stretching vibration of the carbon–carbon double bond ν_C=C_ *cis*-olefinic; a weak band at 1416–9 cm^−1^ assigned to bending in-plane deformation of the olefinic carbon–hydrogen bond δ_C–H_, and a weak band at 722–3 cm^−1^ assigned to scissoring out-of-plane deformation for the olefinic group CH, γ_CH_^25,26^.

With the exception of grape seed oil, both in sunflower oil and canola oil, comparing the intensity of the two characteristic bands typical for olefinic groups: at 3007 cm^−1^ ν_C–H_ olefinic and 1653 cm^−1^ ν_C=C_, FT-IR spectra indicate a lower overall concentration of unsaturated fatty acids of refined oils compared to those cold pressed.

The purpose of this paper was to check if using this analytical method, notable differences between cold pressed and adulterated oils with refined oils by the same type may be observed, and not to quantitatively identify the content in saturated and unsaturated fatty acids (respectively the ratio between them). It may be noticed that the informations provided by the FT-IR spectra cannot be used in establishing adulteration of a cold-pressed oil with its refined one.

### *GC–MS* analysis

*GC–MS* analysis is widely used to determine fatty acid composition of different oils^7,10,23,27–33^.

By the hydrolysis of oils, followed by the derivatization of fatty acids as methyl esters^34,35^, the oil composition of cold pressed (CP) and refined (R) ones was determined by GC–MS (Table 2).

**Table 2:** Fatty oil composition of cold pressed (CP) and refined (R) oils.

Type of oil	Content [mmol/L]				
	Methyl laurate (t_R_ = 19.32 min)	Methyl miristate (t_R_ = 25.31 min)	Methyl oleate (t_R_ = 37.80 min)	Methyl linoleate (t_R_ = 39.09 min)	Methyl linolenate (t_R_ = 41.00 min)
**Coconut**					
CP	41.17	122.85	8.31	3.65	0.00
R	40.51	116.21	4.86	4.37	0.00
**Sunflower**					
CP	2.22	0.00	17.17	56.40	0.00
R	29.63	88.14	7.52	3.59	0.00
**Grapeseed**					
CP	0.00	0.00	22.12	147.61	6.89
R	0.00	0.00	11.42	27.85	2.86
**Canola**					
CP	0.00	0.00	55.13	16.74	7.65
R	0.00	0.00	6.19	4.13	2.85

For the three types of oils rich in unsaturated fatty acids: sunflower, grapeseed and Canola oils, concentrations in oleic acid (ω-9), linoleic acid (ω-6) and linolenic acid (ω-3) were significantly lower in refined oils compared to cold pressed ones, so this method may be used for differentiating a cold pressed oil from a refined one, but it is a complex method, requiring derivatization of oils, and calculation of fatty oil composition from chromatograms.

### Oil colour and metamerism effect

As known, colour sensation results from combining the following factors, which are a lighting source, an object and an observer. The lighting source physically exists, and its spectral energetic distribution may be measured. CIE (International Illuminating Committee) introduced some standard illuminants, among them being CIE A – incandescent light, CIE D65 – white natural light and CIE F2 – cold fluorescent.

Two coloured surfaces can stimulate all three centers of excitation of the eye to the same extent under a specific illumination, but not under a different light source, appearing identical in the first case, and different in the second case, the phenomenon being called metamerism^36,37^.

Food oils may be observed under different illuminants, depending on where they are displayed in the supermarkets, resulting different colour parameters (Table 3), fact that indicates that food oils present the metamerism effect.

**Table 3:** Colour parameters of all refined (R) and cold pressed (CP) oils for different illuminants.

Illuminant	Canola oil	L*	a*	b*
D65	R	88.89	− 2.34	8.86
	CP	83.99	3.63	33.02
A	R	89.23	0.12	8.39
	CP	86.31	6.32	36.38
F2	R	89.16	− 1.68	10.07
	CP	86.33	0.81	36.97

Illuminant	Coconut oil	L*	a*	b*
D65	R	90.34	− 0.78	2.91
	CP	90.96	− 0.26	0.61
A	R	90.46	0.07	2.76
	CP	90.72	− 0.06	0.56
F2	R	90.44	− 0.6	3.33
	CP	90.97	− 0.22	0.71

Illuminant	Sunflower oil	L*	a*	b*
D65	R	90.5	− 0.88	2.29
	CP	90.23	− 1.39	14.35
A	R	90.56	− 0.18	2.09
	CP	91.02	0.92	15.29
F2	R	90.56	− 0.66	2.64
	CP	90.95	− 1.38	15.51

Illuminant	Grapeseed oil	L*	a*	b*
D65	R	90.51	− 2.51	7.24
	CP	87.2	− 1.55	23.23
A	R	90.72	− 0.38	6.64
	CP	88.47	1.9	24.53
F2	R	90.7	− 1.77	8.29
	CP	88.38	− 1.67	25.37

**Figure 1:**
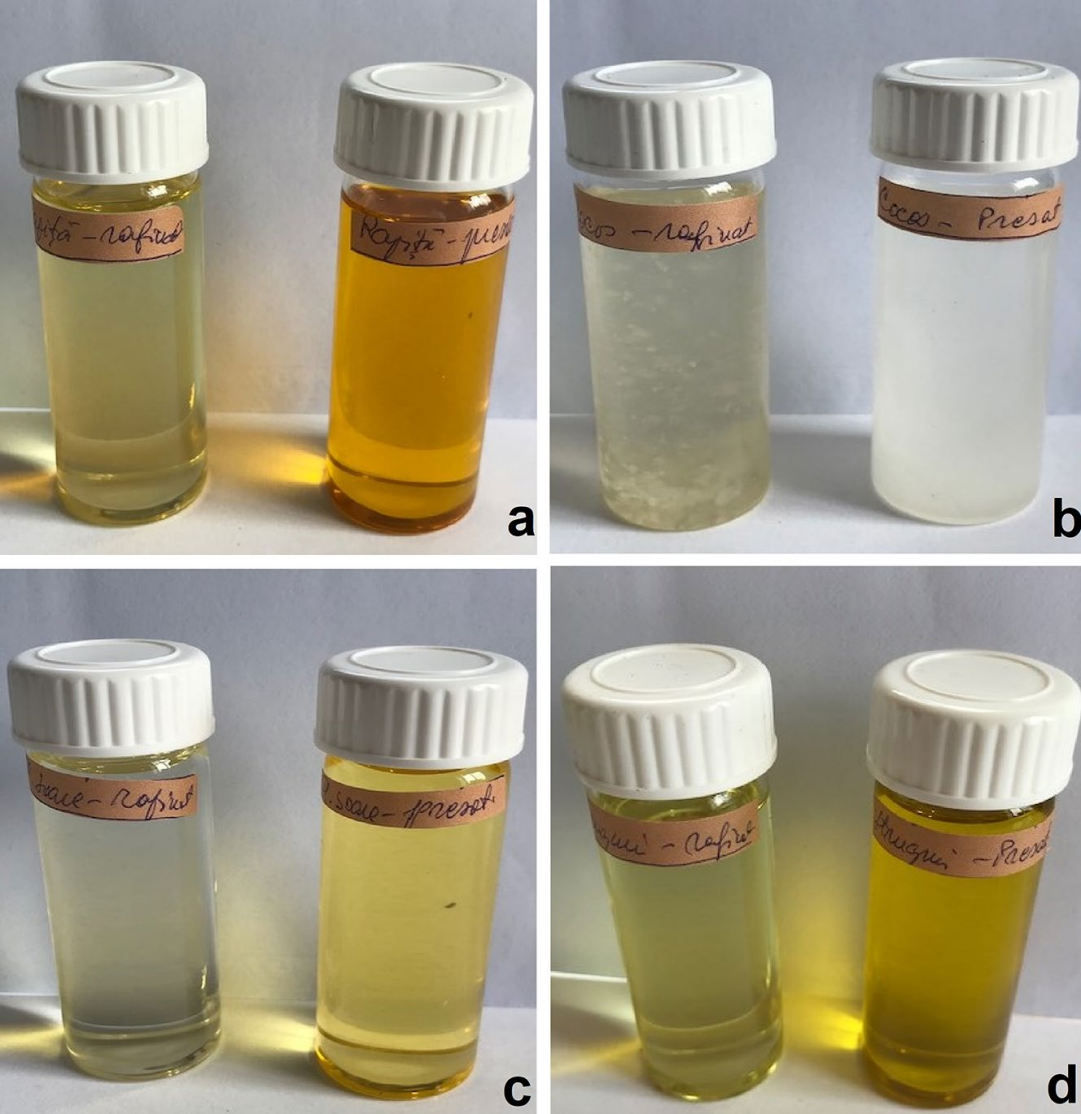
Visual colour difference between refined (left) and cold pressed (right) oils: (**a**) Canola; (**b**) coconut; (**c**) sunflower; (**d**) grapeseed.

When using the D65 illuminant (assimilated to natural open-air daylight), all the refined oils except the coconut oil, present higher values for lightness than cold pressed ones, which may also be observed by the naked eye (Fig. 1).

Regarding the a* parameter value, for the cold pressed Canola oil it is in the red domain but for the refined one is in the green domain; for all the other cold pressed and refined oils, a* parameter value is in the green domain. The values for the a* parameter are three times higher for the refined coconut oil than for the cold pressed one, two times higher for the refined grapeseed oil than for the cold pressed one, and double for the cold pressed sunflower oil as compared to that of the refined one.

All oils have the b* parameter value in the yellow domain, presenting great differences in value between cold pressed oils and the refined ones: four times higher for the cold pressed Canola oil, six times higher for the refined coconut oil, seven times higher for the cold pressed sunflower oil, and three times higher for the cold pressed grapeseed oil.

### Colour study of adulteration of food oils with the refined ones

Colour measure being a good method for differentiating cold pressed oils and refined ones we propose it as a rapid detection method for adulteration of cold pressed oils with their refined versions in different proportions (mass%).

**Figure 2:**
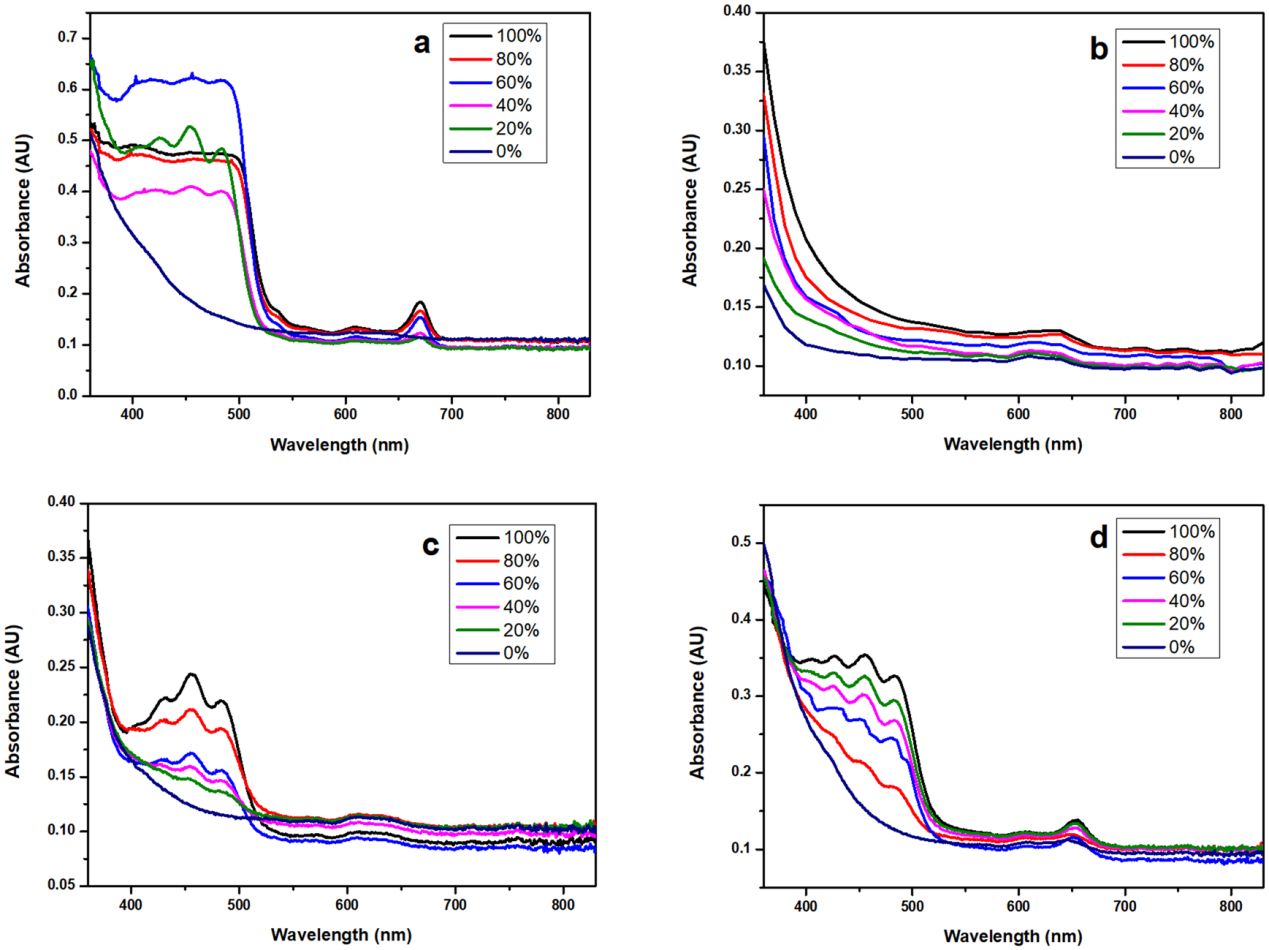
Absorbance spectra of cold pressed oils adulterated with refined ones (percentage of cold pressed oil): (**a**) Canola; (**b**) coconut; (**c**) sunflower; (**d**) grapeseed.

**Figure 3:**
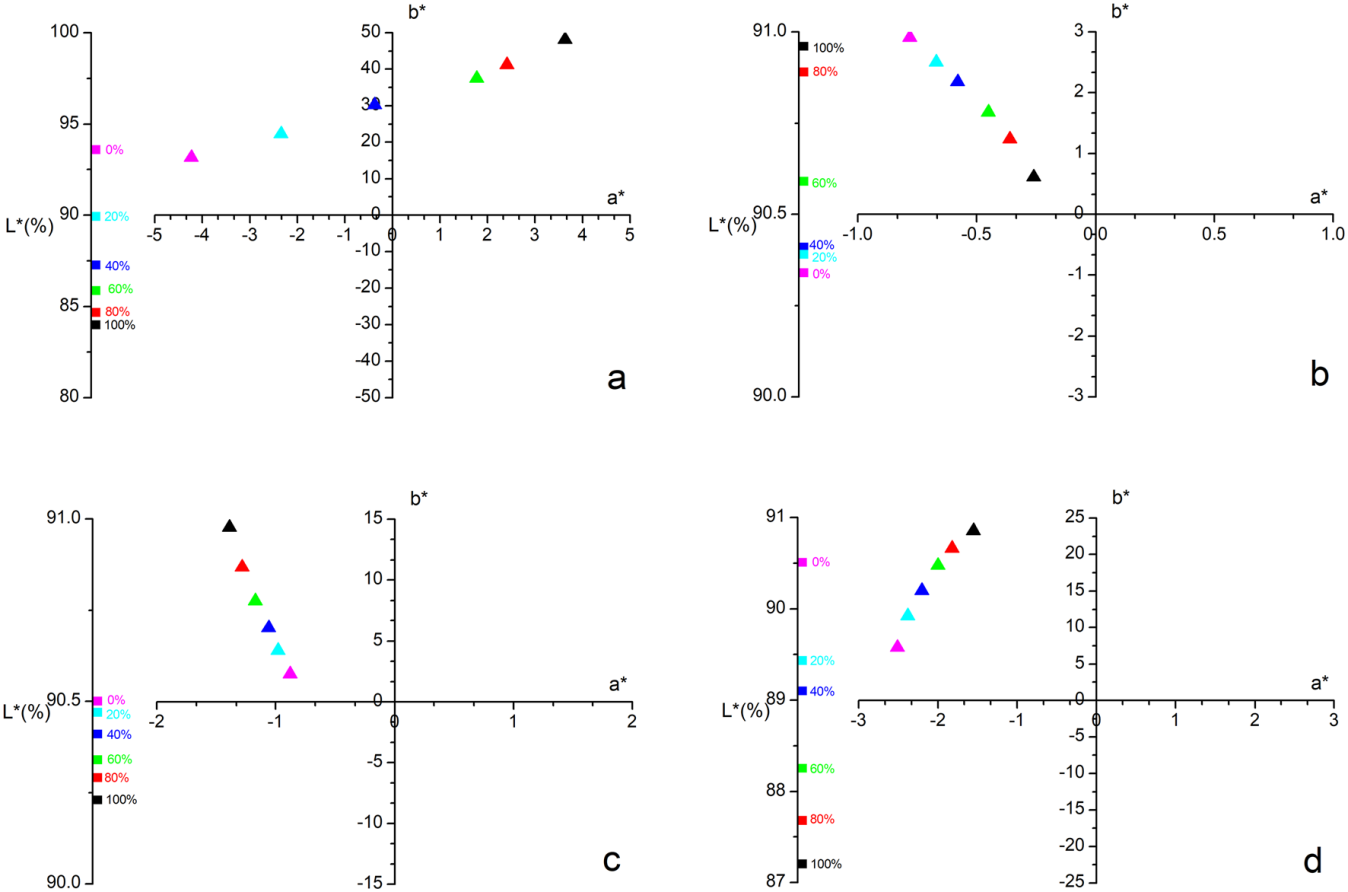
CIE L*a*b* parameters of cold pressed oils adulterated with refined ones (percentage of cold pressed oil): (**a**) Canola; (**b**) coconut; (**c**) sunflower; (**d**) grapeseed.

Colour analyses of this adulteration reveal that the absorbance spectra (Fig. 2) and the CIE L*a*b* parameters (Fig. 3) differ with the cold pressed oil content in the mixture.

Absorbance spectra of cold pressed oils present a maximum at about 650 nm for all oils, and, except coconut oil, triplets at 450–500 nm^36^. These maxima do not appear in any of the refined oils. When adulterating cold pressed oils with refined ones, these maxima appear on the absorbance spectra, but they fade out as the percentage of refined oil adulteration increases.

**Figure 4:**
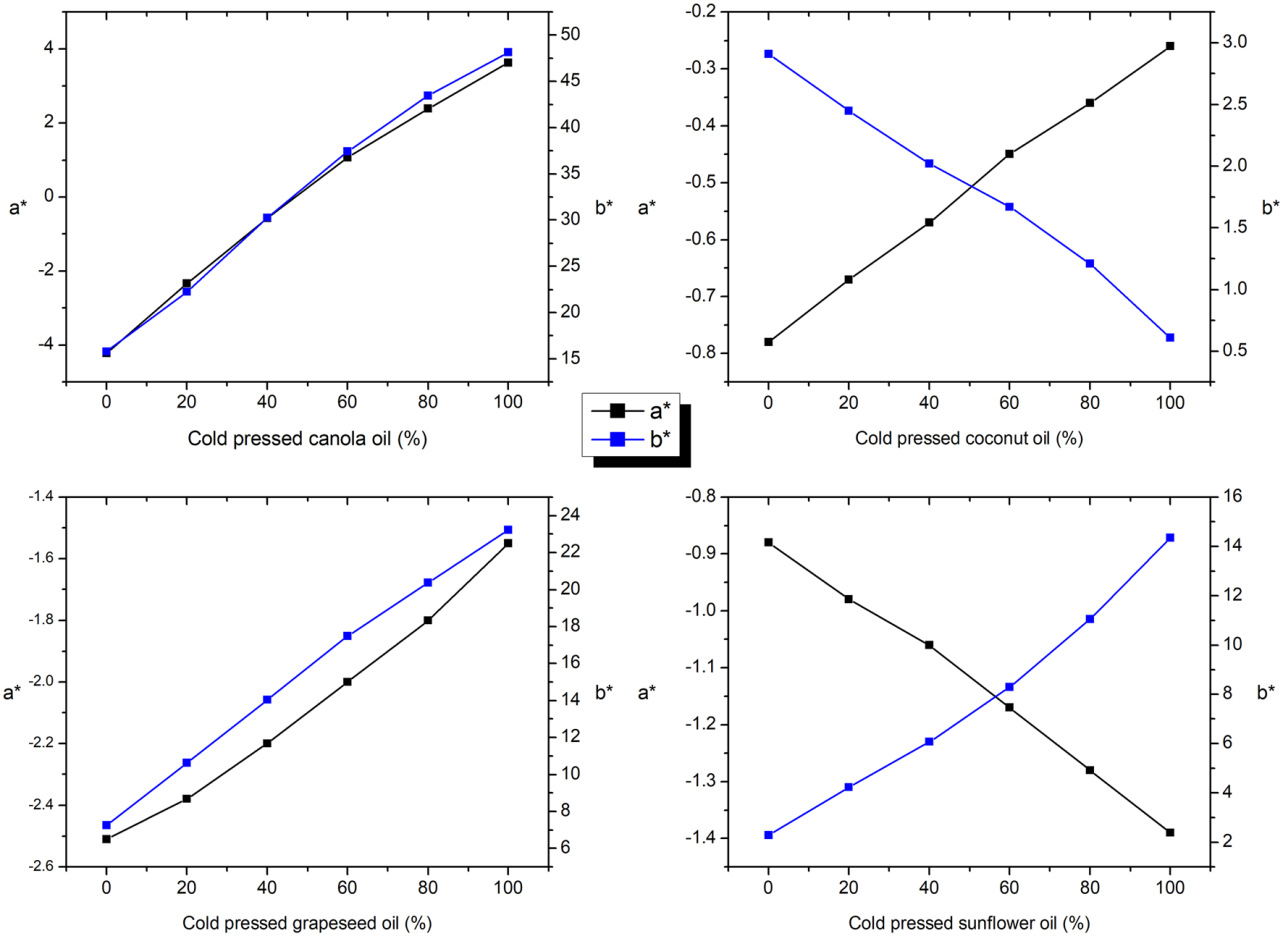
Dependence of a* and b* parameters on cold pressed oil content (mass%).

CIE L*a*b* parameters of all oil mixtures (Fig. 3) show that the lightness increases when adding the refined oil to the cold pressed one, as expected, except for the coconut oil (Fig. 1). The a* and b* parameters have a linear dependence for all the studied oils (Fig. 4), with the equations presented in Table 4. The correlation coefficient R^2^ is 0.99 for all equations.

**Table 4:** Dependence equations for a* and b* on cold pressed oil content (mass%) in the mixtures.

Cold pressed oil	a*		b*	
	Intercept	Slope	Intercept	Slope
Canola	− 3.95 ± 0.11	0.079 ± 0.003	16.25 ± 1.32	0.332 ± 0.002
Coconut	− 0.78 ± 0.07	0.005 ± 0.001	2.92 ± 0.91	− 0.022 ± 0.012
Sunflower	− 0.87 ± 0.05	− 0.005 ± 0.002	1.79 ± 0.4	0.119 ± 0.021
Grapeseed	− 2.55 ± 0.7	0.010 ± 0.0005	7.45 ± 0.63	0.161 ± 0.0005

With these established equations, the amount of cold pressed oil in a product found in the supermarkets may be calculated, after determining the CIE L*a*b* parameters by UV–Vis spectroscopy.

## Materials and methods

Four different cold pressed oils and their refined versions were purchased from the Romanian market: coconut oil, sunflower oil, grapeseed oil and Canola oil, for a period of three years. Every year we purchased all oils under the same brand names, in order to compare the results. Because the properties of the oils were similar, in this paper we only presented one example for each oil purchased in the last year of the research. The chemicals used in this study were of analytical grade.

Density was determined using pycnometer method.

Refraction index was determined using an Abbe-Zeiss refractometer.

Acid number was determined according to ISO 660: 2009 method.

Peroxide value was determined according to ISO 3960: 2017 method.

Viscosity was determined using a Brookfield CAP 2000^+^ L viscosimeter.

FT-IR spectra were recorded by the film working technique, with KBr pellets, using a Jascow FT-IR-430 spectrophotometer, at a resolution of 4 cm^−1^.

The chromatograms and the mass spectra corresponding to the chromatographic peaks were recorded using a GC–MS Thermo Scientific System TRACE 1310, ITQ 1100 Ion Trap MS. The column used was TG-WAXMS 30 × 0.25 mm × 0.5 μm, Thermo Scientific. Temperature program: 80 °C (1 min) 150 °C (0.5 min) - 240 °C (0.5 min)/3 °C, 240–300 °C (2 min)/7 °C/min. Injector temperature 250 °C, helium flow rate 1 mL/min. MS parameters were: transfer line temperature at 310 °C, reading range 30–700 m/z. Hexadecane was used as internal standard.

Colour analysis was conducted using a Cary-Varian 300 Bio UV–VIS colourimeter with integrating sphere, using a Spectralon standard and three illuminants: D65, A and F2. All colour data were expressed by L*, a*, b* coordinates*,* where L* corresponds to lightness; a* corresponds to the transition from green (− a*) to red (+ a*); and b* corresponds to the transition from blue (− b*) to yellow (+ b*).

### Additional Information

In order to verify our theory, colour analysis was also performed on the same four different cold pressed oils (coconut oil, sunflower oil, grapeseed oil and Canola oil) and their refined versions, which were purchased under different brand names. All the results are presented in the Supplementary Information.

The proposed equations for the a* and b* parameters for the oils purchased under the same brand names are similar to the ones proposed for the oils purchased under different brand names.

## Conclusions

This study describes some methods for differentiating cold pressed food-grade oils from the refined ones. Classical physico-chemical properties, such as density, viscosity, acid index, saponification index, peroxide value, or refractive index present very similar values for the tested cold-pressed oils and their refined versions, so these physico-chemical parameters may not be successfully used for distinguishing a cold pressed oil from a refined one, and for the adulteration detection purposes. *FT-IR* cannot be used in establishing adulteration of cold-pressed oil with a refined one, because there are no noticeable differences between FT-IR spectra of the cold–pressed oils and of their refined versions. *GC–MS* analysis may be used for differentiating a cold pressed oil from a refined one, but it is a complex method, requiring derivatization of oils, and calculation of fatty oil compozition. Colour difference between the cold pressed oils and the refined ones may be visually appreciated and determined by UV–Vis spectroscopy. For this reason, this last investigation technique was proposed as a rapid method for appreciating adulteration of cold pressed oils with refined ones. When adulterating cold pressed oils with refined ones, the maxima in the absorbance spectra fade out as the percentage of refined oil adulteration increases.

Regarding CIE L*a*b* parameters, dependence equations for a* and b* on cold pressed oil content (mass%) were proposed, that may be used to calculate the amount of cold pressed oil in a product.

## Supplementary information

Supplementary Information.
