# Supplementary material for: Rapid adulteration detection of cold pressed oils with their refined versions by UV–Vis spectroscopy
Source: Sci Rep. 2020 Sep 30;10:16100. doi: 10.1038/s41598-020-72558-7 (PMC7528079; doi:10.1038/s41598-020-72558-7)
Supplement: Supplementary file 1 — Supplementary Information. [file 41598_2020_72558_MOESM1_ESM.docx]

**Supplementary Material**

Adulteration assessment by colour study of edible oils mixed with their refined versions was performed for oils (coconut oil, sunflower oil, grapeseed oil and Canola oil) purchased from the Romanian market under different brand names.

Colour analysis was conducted using the Cary-Varian 300 Bio UV-VIS using a Spectralon standard and the D65 illuminant. All colour data were expressed by *L*, a*, b** coordinates.

The mixtures for all oils used in these investigations (Fig. 1SP) reveal that the luminosity increases with the percentage of the refined versions, except for the coconut oil, that has an opposite behaviour.


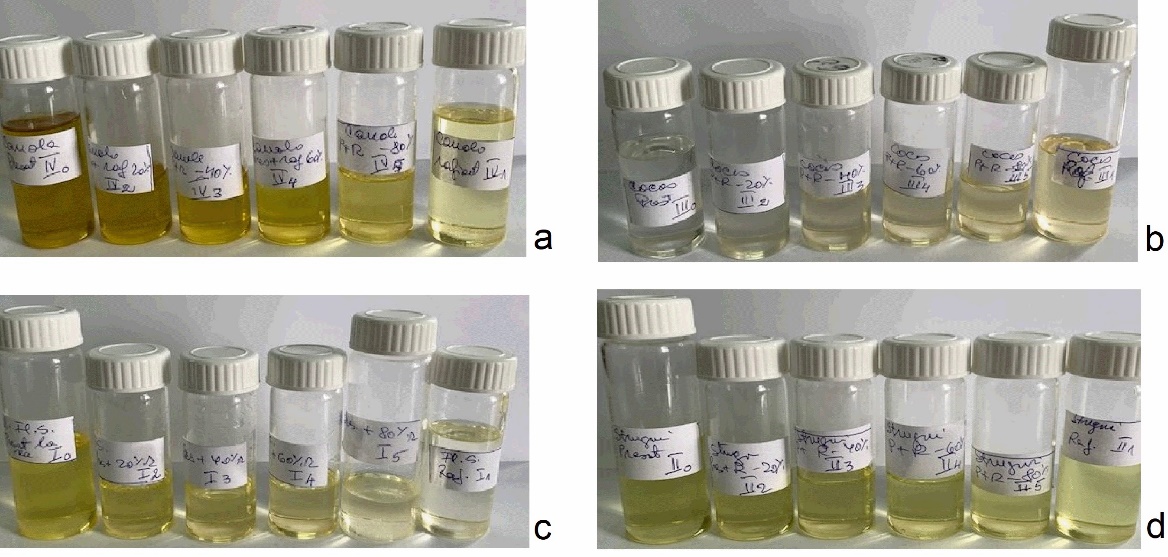


**Figure 1SP.** The colour of the cold pressed oils (first left) adulterated with their refined versions, (percentage of the refined oil increasing): a-Canola; b-coconut; c-sunflower; d-grapeseed

Colour analyses of this adulteration reveal that the absorbance spectra (Fig. 2SP.) and the CIE L*a*b* parameters (Fig. 3SP.) differ with the concentration of the cold pressed oil in the mixture.


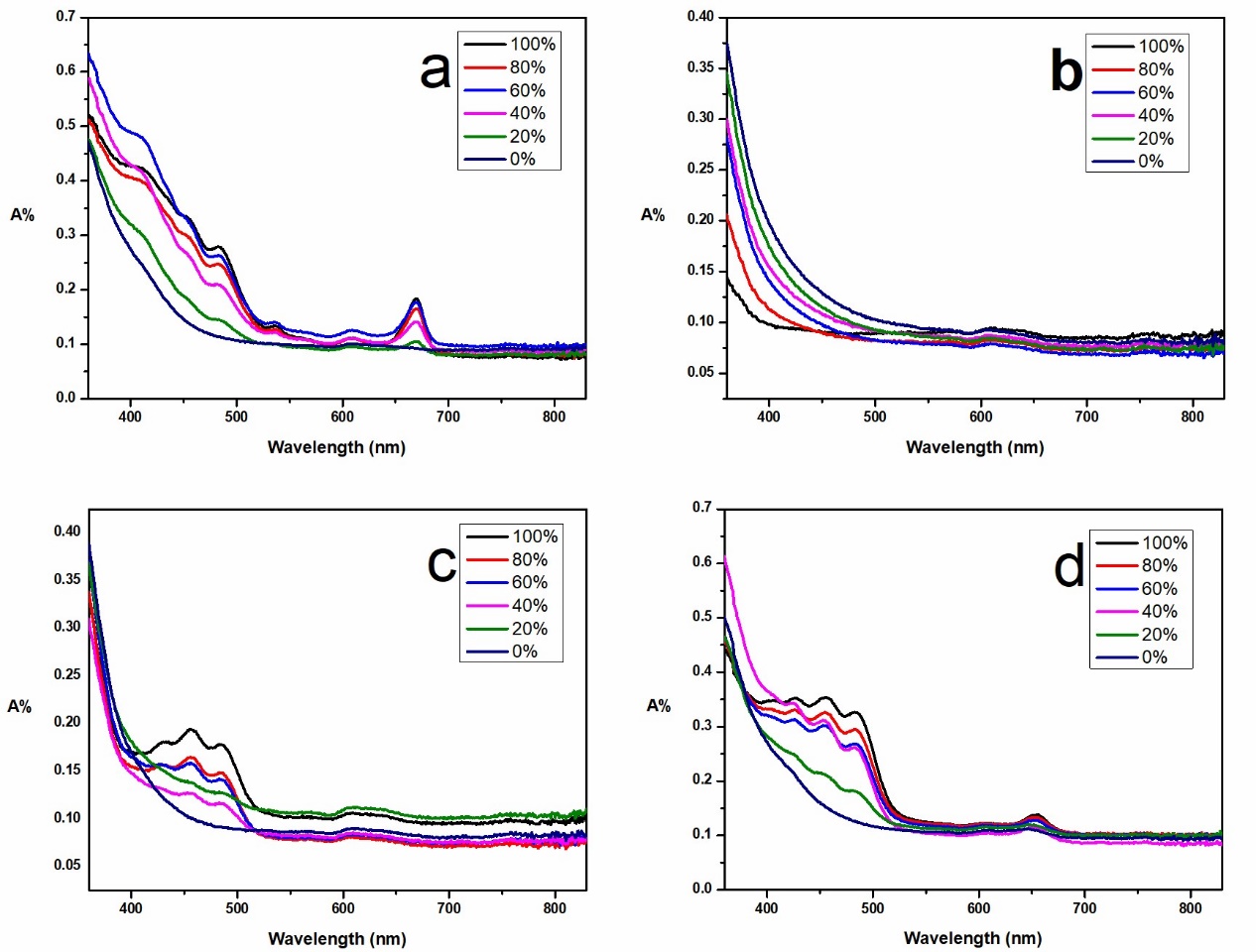


**Figure 2SP.** Absorbance spectra of cold pressed oils adulterated with their refined versions (percentage of cold pressed oil) - a-Canola; b-coconut; c-sunflower; d-grapeseed

Absorbance spectra of cold pressed oils present a maximum at about 650 nm for all oils, and, except coconut oil, triplets at 450-500 nm. These maxima do not appear in any of the refined oils. When adulterating cold pressed oils with refined ones, these maxima appear on the absorbance spectra, but they fade out as the percentage of refined oil adulteration increases.

CIE L*a*b* parameters of all oil mixtures (Fig. 3SP.) show that the lightness increases when adding refined oil to the cold pressed one, as expected, except for the coconut oil (Fig. 1SP).


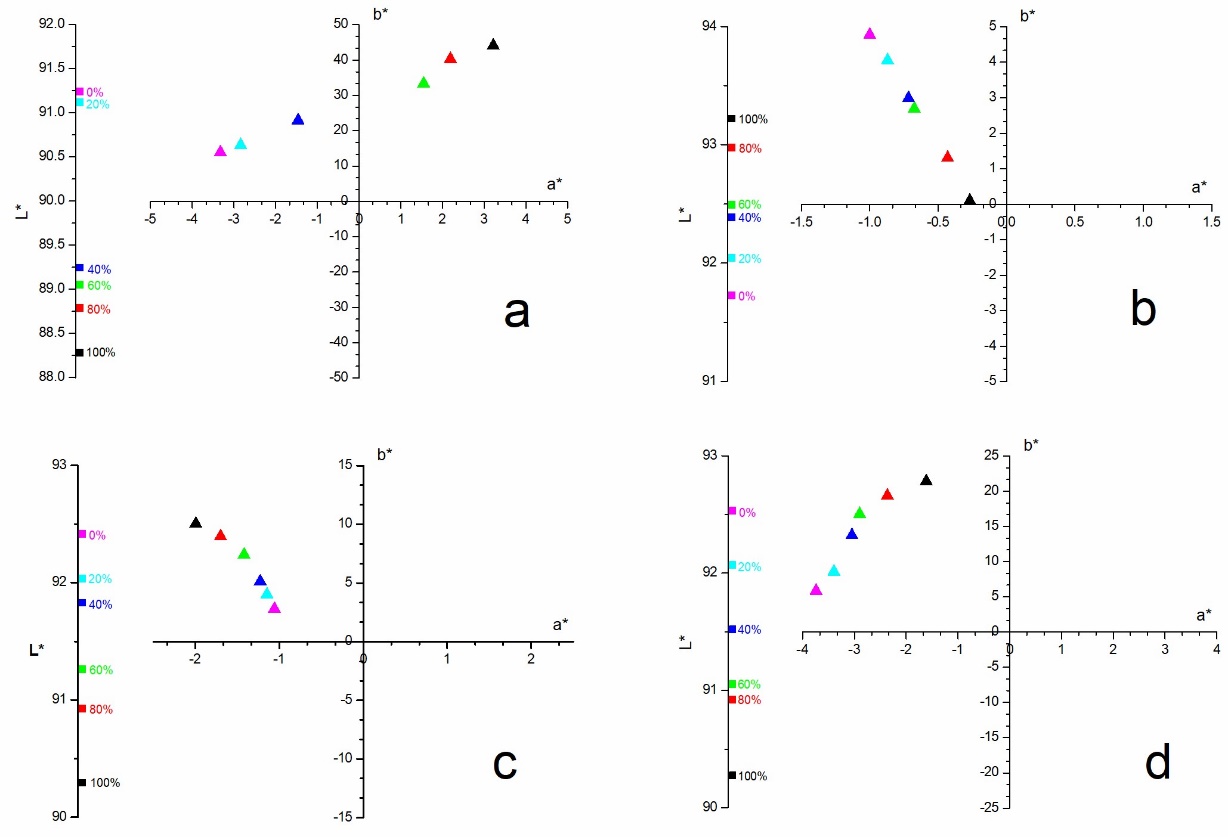


**Figure 3SP.** CIE L*a*b* parameters of cold pressed oils adulterated with refined ones (percentage of cold pressed oil) - a-Canola; b-coconut; c-sunflower; d-grapeseed

The *a** and *b** parameters have a linear dependence with the cold pressed oil concentration for all the studied oils, having the equations presented in Table 1SP.

| **Cold pressed oil** | ***a** = m + n [C]** | ***b** = m + n [C]** |
| --- | --- | --- |
| Canola | *a** = -3.739 + 0.0726 [C] | *b** = 13.6179 + 0.3363 [C] |
| Coconut | *a** = -0.9182 + 0.0071 [C] | *b** = 4.9308 – 0.0455 [C] |
| Sunflower | *a** = -0.9565 - 0.0093 [C] | *b** = 2.5775 + 0.0762 [C] |
| Grapeseed | *a** = -3.8335 + 0.0108 [C] | *b** = 6.1984 + 0.1618 [C] |

**Table 1SP.** Dependence equations for *a** and *b** on cold pressed oil concentration ([C])

The proposed equations of the *a** and *b** parameters for the oils purchased from the same brand names are similar to the ones proposed for the oils purchased from different brand names.
